# ShapoGraphy: A User-Friendly Web Application for Creating Bespoke and Intuitive Visualisation of Biomedical Data

**DOI:** 10.3389/fbinf.2022.788607

**Published:** 2022-07-04

**Authors:** Muhammed Khawatmi, Yoann Steux, Saddam Zourob, Heba Z. Sailem

**Affiliations:** Institute of Biomedical Engineering, Department of Engineering, University of Oxford, Oxford, United Kingdom

**Keywords:** microscopy, multiplexed imaging, morphology, glyph-based visualisation, high dimensional data, graph editor, single cell data, science communication

## Abstract

Effective visualisation of quantitative microscopy data is crucial for interpreting and discovering new patterns from complex bioimage data. Existing visualisation approaches, such as bar charts, scatter plots and heat maps, do not accommodate the complexity of visual information present in microscopy data. Here we develop ShapoGraphy, a first of its kind method accompanied by an interactive web-based application for creating customisable quantitative pictorial representations to facilitate the understanding and analysis of image datasets (www.shapography.com). ShapoGraphy enables the user to create a structure of interest as a set of shapes. Each shape can encode different variables that are mapped to the shape dimensions, colours, symbols, or outline. We illustrate the utility of ShapoGraphy using various image data, including high dimensional multiplexed data. Our results show that ShapoGraphy allows a better understanding of cellular phenotypes and relationships between variables. In conclusion, ShapoGraphy supports scientific discovery and communication by providing a rich vocabulary to create engaging and intuitive representations of diverse data types.

## 1 Introduction

Biomedical imaging generates large amounts of data capturing biological systems at different scales ranging from single molecules to organs and organisms (Walter et al., 2010). Inspection of individual images is not feasible when hundreds of images are acquired, particularly when they are composed of multiple layers, channels, or planes. Automated image analysis allows quantifying image data resulting in large multiparametric datasets (Sero et al., 2015; Natrajan et al., 2016). Effective data visualisation is essential for interpreting analysis results and unleashing the hidden patterns locked in image data (Heer et al., 2010; Cairo, 2013).

Intuitive representations can improve the effectiveness of visualisation tools as they support identifying and understanding the complex relationships in image data. By intuitive we mean that the depicted representations are semantically relevant where the used visual channel resembles the concept or the represented phenotypic feature. For example, it is easier to associate measurements of cell size to the size of the object and the protein levels to the colour of the object. This has many advantages especially when multiple variables are plotted simultaneously. First, the pictorial representation facilitates remembering and interpreting the data. Second, the natural mapping between the measured objects and the representation makes it easier to investigate the relationship between the measured variables.

Visualising complex imaging data has been mostly limited to general-purpose tools that do not take into account the structural nature of image data. Due to their scalability to a large number of data points, heat maps and dimensionality reduction, such as UMAPs and t-SNE, are the most used approaches for visualising high dimensional data, including image-based measurements (McInnes et al., 2018). Several methods have been developed for visualising bioimage data with an emphasis on interactive linkage of raw image data, cell features, and identified quantitative phenotypes using linked scatter plots combined with supervised and unsupervised learning approaches including t-SNE plots. These include Facetto, histoCAT, and mineotaur (Antal et al., 2015; Schapiro et al., 2017; Krueger et al., 2020). ImaCytE (Somarakis et al., 2019) is another tool for visualising multiplexed image cytometry data that takes the interactive aspect a step further by developing custom two-layered pie charts to represent the proportion of different phenotypes. While these tools are useful in interactive and data exploration tasks, they heavily rely on the user interpretation of identified phenotypes based on the appearance of a handful of cells which can be a subjective and daunting task. Therefore, new visualisation techniques for representing multiparametric image data are desperately needed to aid data analysis and result interpretation.

Glyph-based visualisation is another approach to visual design where quantitative information is mapped to illustrative graphics referred to as glyphs. They provide a flexible way of representing multidimensional data (Ropinski et al., 2011; Borgo et al., 2013; Fuchs et al., 2017). For example, we have previously developed PhenoPlot, a glyph-based visualisation approach that plots cell shape data as cell-like glyphs (Sailem et al., 2015). PhenoPlot was built as is a MatLab toolbox and incorporates two ellipsoid glyphs to represent the cell and nucleus. It uses a variety of visual elements such as stroke, colour and symbols to encode up to 21 variables. The key focus of PhenoPlot is to allow for natural data mapping by selecting graphic features that resemble data attributes. For instance, the extent that a jagged border around the cell ellipse can be used to represent the irregularity of cell shape, and the proportion of “x” symbols filling the cell ellipse can be mapped to endosome abundance. However, the shape configuration in PhenoPlot is limited to two ellipse-shaped objects and the feature mapping is hard-coded which does not accommodate the diversity of biomedical images data.

To support knowledge discovery tasks from microscopy data, we propose a new framework for creating glyph-based representations by combining geometrical shapes that can systematically encode several predefined visual elements. We implemented this framework as a user-friendly web interface that can automatically and swiftly map data to the created glyph representations. To our knowledge, ShapoGraphy is the first method that allows creating new glyph-based visualisation by combining different shaped objects and custom mapping of their properties, such as colour, symbols, stroke, and dimensions, to data attributes. The user can choose from a basic set of shapes or draw their own. The effectiveness and utility of ShapoGraphy are illustrated by using various image datasets where we show that it facilitates the understanding of cellular phenotypes and interactive exploration of the data. This includes multiplexed image data where single cell activities of tens of proteins are measured simultaneously. In summary, ShapoGraphy allows the users to construct an infinite number of glyph-based representations in order to generate a quantitative and intuitive visualisation to aid pattern recognition from multiparametric data.

## 2 Methods

### 2.1 Design and Concept of ShapoGraphy

**FIGURE 1:**
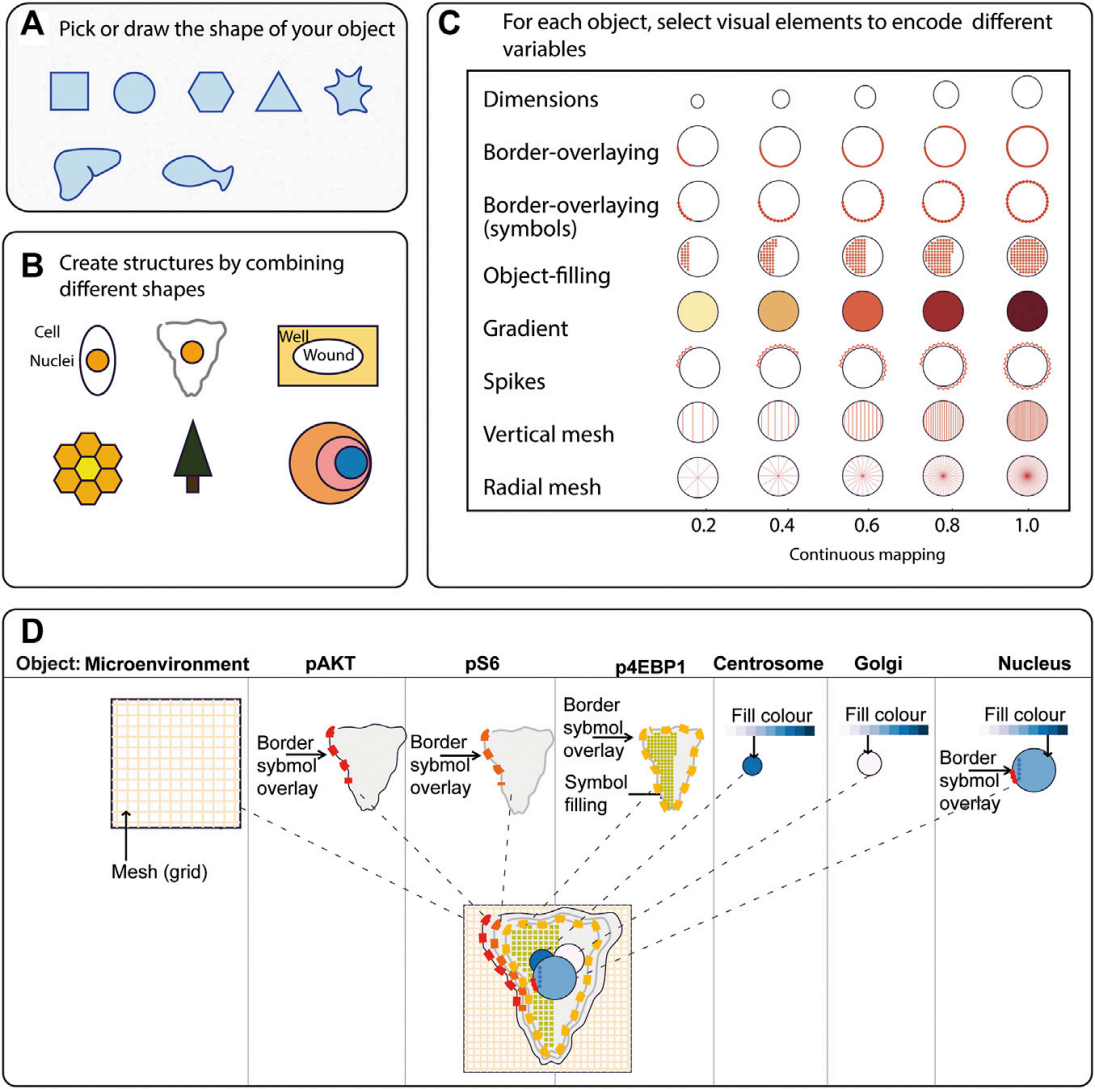
ShapoGraphy provides a highly flexible framework for creating glyph-based visualisations. **(A)** Example of object shapes that can be created using Shapography. **(B)** Shapes can be combined to create structures that resemble the measured phenomena. **(C)** Various visual elements are defined for each object and can be selected by the user to encode several variables. **(D)** An example of how objects can be combined to represent a wide range of phenotypic information.

**FIGURE 2:**
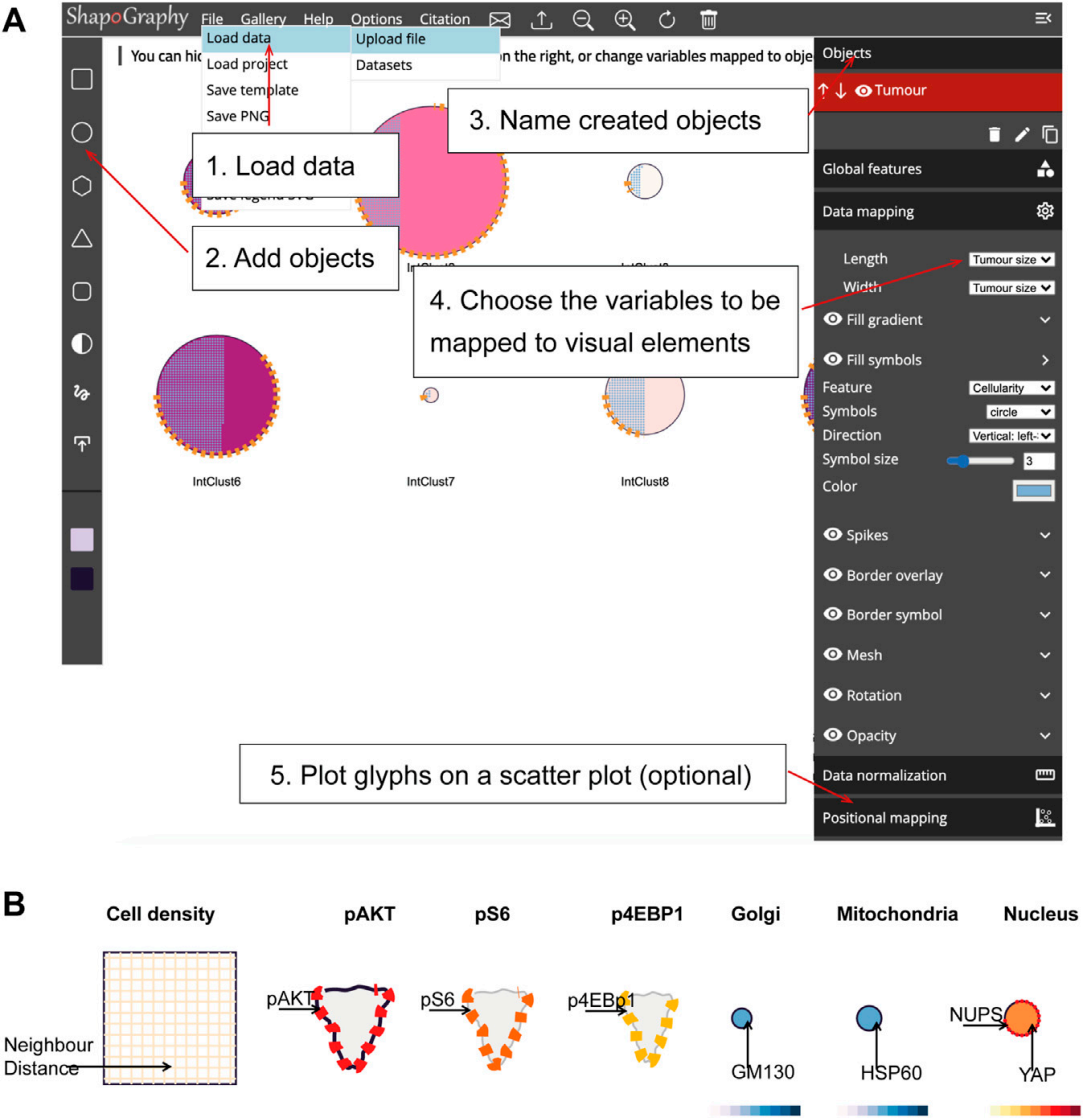
ShapoGraphy user interface. **(A)** ShapoGraphy allows users to interactively construct and customise their plots using a flexible graphical user interface. The user 1) uploads the data from the file menu 2) creates objects 3) customises their properties 4) maps the selected object properties to the variables in the dataset. Positional mapping can be used to position the created objects in a scatter plot based on selected data variables. **(B)** Legend is generated automatically by ShapoGraphy where different objects are shown seperately and variables mapped to the different visual elements for each object are labelled. Objects names chosen by the user are shown in bold. All other labels are the variable names that are mapped to the object properties or depicted marks.

To generate a quantitative pictorial representation of phenotypic data we created ShapoGraphy; a user-friendly web application (Figures 1A–D, 2A). ShapoGraphy maps data to visual properties of shapes where multiple shapes can be combined to define a biological structure. For example, a squared-shaped object can be used to represent cell context, epithelial cell shape can be represented using a square for the cell body and a circle for the nucleus. We call such a configuration a template and provide multiple templates to represent a variety of microscopy data. A new template can be created by combining different shapes. The users have the option of selecting from a collection of predefined geometrical shapes or drawing their own. For example, the user can draw a cell or organ shape. The objects can be positioned relative to each other to create the desired structure (Figure 1D). ShapoGraphy is highly customisable where the property of any object in the template, such as colour, size or opacity, can be changed.

We developed various encodings that allow mapping continuous quantitative data to shapes by using different visual elements (Figure 1C). These include dimensions, size, and colour that are commonly used for visualising data. For the fill gradient element, we employed well-established colour maps from ColorBrewer (Brewer, 2022). We have previously proposed novel visual elements, such as partial overlaying the object outline or filling the object with symbols proportional to the variable value (Sailem et al., 2015). We introduce new features in ShapoGraphy, such as the mesh density (horizontal, vertical or grid), opacity, and rotation angle (Figure 1C). The use of various glyph shapes, positions and visual elements allows designing abstract and intuitive representations of a broad range of structures investigated in biomedical imaging to assist in understanding, summarising, and communicating results (Figure 1D). This type of design gives the user high flexibility when it comes to constructing new visual encodings that are more intuitive and engaging.

### 2.2 ShapoGraphy User Interface

We adopted a modular design that resembles other graphic design software such as Adobe Illustrator. Data import, saving results, figure export and other auxiliary functionalities such as viewing the data in a heat map or t-SNE plots are available from the top menu (Figure 2). Once a dataset is uploaded, the user can add various shapes from the left menu. This includes drawing a custom shape using the “draw shape” icon which opens a small canvas that the user can draw on. For this option, the user needs to draw the shape in one stroke as many elements, such as border symbols or overlay, will be mapped to the object outline. A list of the added shapes will appear on the right menu. The user can modify the name of each object using the pencil icon at the bottom of the objects list so that they can be easily identified. The user can also duplicate an object which can be useful to generate a new object with exact feature mapping or when a custom shape is used. The objects are laid on top of each other as layers. The object layer order can be modified using the upward and downward arrows on the left of the object name. For example, the nucleus should be positioned after the cell object, as it will be concealed otherwise. The object location can be changed from the Global Features sub-menu or by dragging and dropping the object in the canvas.

For each object, we recommend selecting visual channels in such a way that they metaphorically resemble the measured concepts. Different symbols can also be used to distinguish different variables. The user can customise the visual appearance of these channels and the variables that are bound to them from the Data Mapping sub-menu. For example, for “Symbol filling” or “Border symbol” elements, the user can choose from the following symbols: {✕, *, -, •, **□, ▟**} and specify their colour and size (Table 1). For the Mesh element, the user can choose vertical, horizontal, radial, grid-like or randomly oriented mesh (Figure 1C). The user can also specify the stroke size of the mesh and the colour of the mesh lines.

**TABLE 1:** Customisable properties of ShapoGraphy elements.

Visual element	Static properties
Length	No additional properties
Width	No additional properties
Fill gradient	Colour map
Fill symbols	Symbol: { ✕, *, -, •, **□, ◷**}
	Fill direction: left- > right, right- > left, top- > bottom, bottom- > top
	Symbol colour
	Symbol size
Spikes	Stroke size
	Spike density
	Colour
Border overlay	Stroke size
	Stroke colour
Border symbol	Symbol: { ✕, *, -, •, **□, ◷**}
	Symbol colour
	Symbol Size
Mesh	Orientation {vertical, horizontal, radial, grid, random}
	Colour
	Stroke size
Rotation	No additional properties
Opacity	No additional properties

To facilitate the exploration of design space in ShapoGraphy, we offer a hide/show functionality of each of the objects or data-symbol mappings through the eye icon on the left of each object or element. We found this functionality very useful when assessing interactions between objects, decluttering the representation or determining relevant features.

On the right menu, there are also options for data normalisation which is discussed in Section 2.4 and positional mapping of Shape Glyphs in 2D dimensional space.

We employ pagination to deal with a large number of data points. The user has the option to display more objects on the same page or browse them in multiple pages. This can be useful if combined with sorting functionality in the Positional Mapping sub-menu.

### 2.3 Legend

**FIGURE 3:**
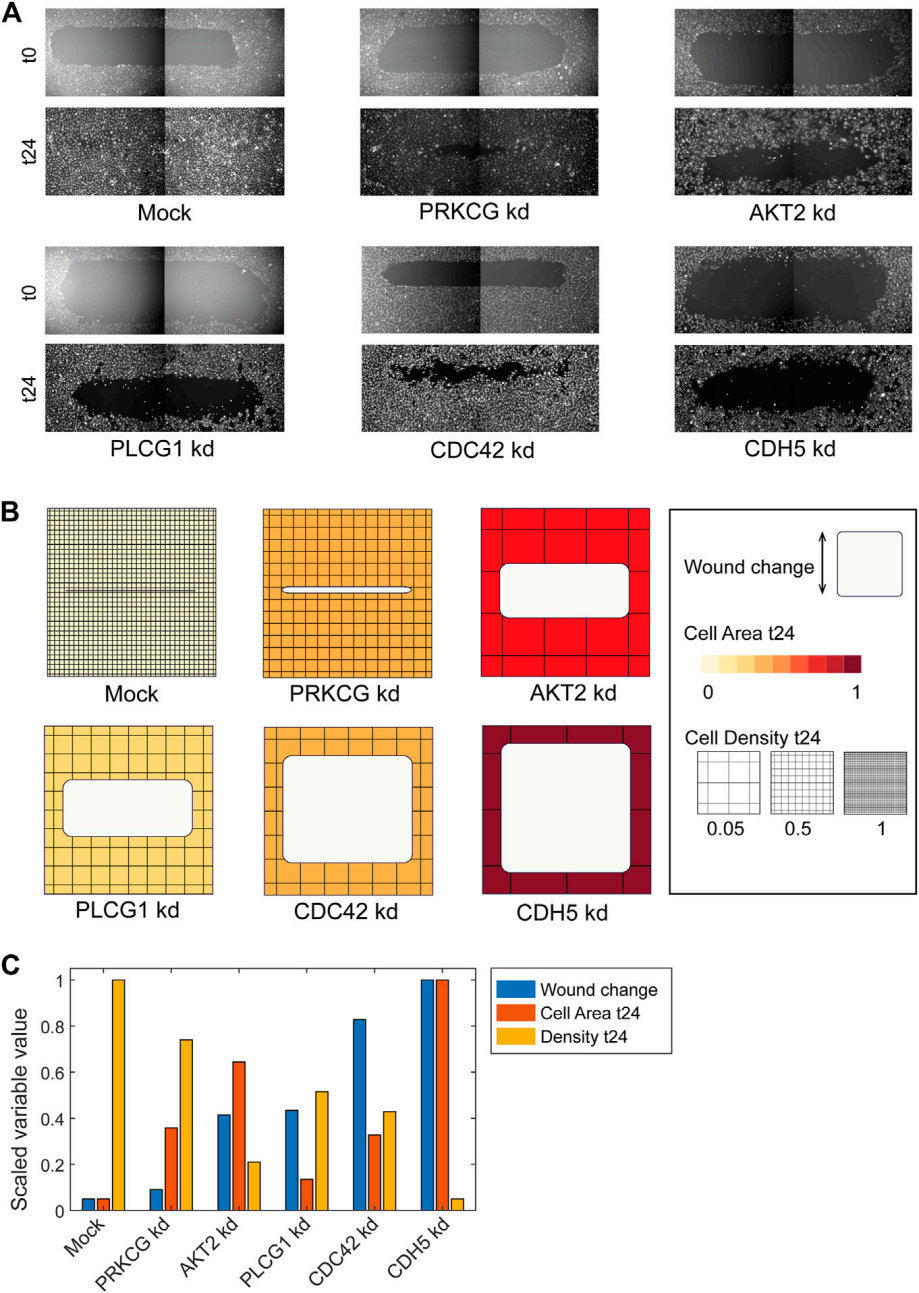
Using ShapoGraphy to represent wound healing data. **(A)** Image data capturing the effect of various gene depletions on human lymphatic endothelial cells ability to migrate into scratch wounds [time-point 0h (t0) and 24h (t24)]. **(B)** Intuitive representation of wound area and cell number measurements using ShapoGraphy based on data in **(A)**. The outer square represents the well where lighter red hues indicate lower cell area while higher red hues indicate higher cell area. Cell number is mapped to grid density. The height of the inner square represents the normalised change in wound area. **(C)** Representation of the same data in **(B)** using a bar chart where numerical data are mapped to the bars’ length

**FIGURE 4:**
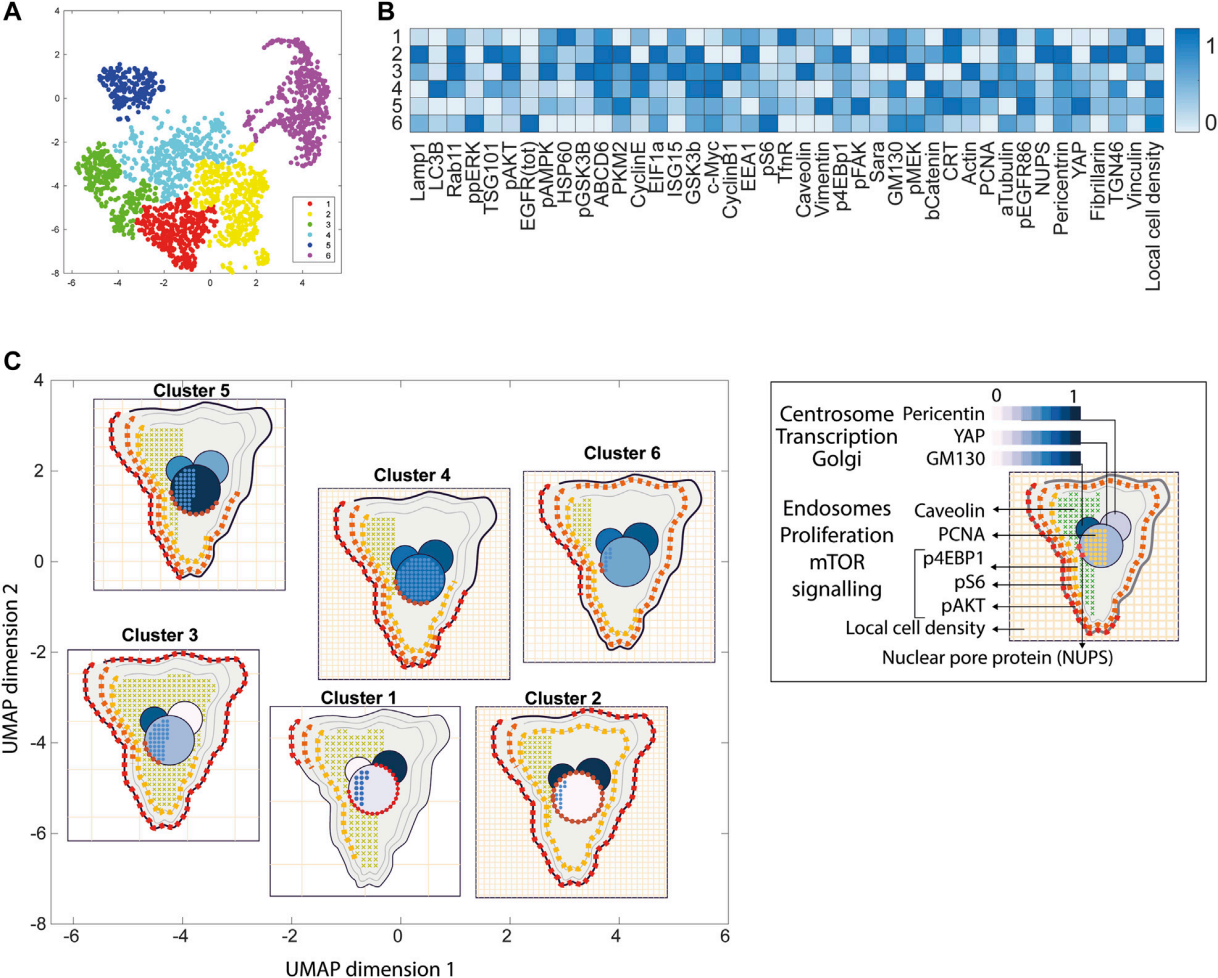
ShapoGraphy allows interpreting multiplexed single-cell data. **(A)** UMAP projection of 2000 single HeLa cells. **(B)** Representation of average values of 40 markers as well as local cell density for each identified cluster using a heat map. **(C)** Representation of selected features from C using ShapoGraphy where the shapes graphs are placed at centre of the cluster.

The legend can be viewed from the top menu. Creating a legend for the resulting composite glyph can be challenging as we do not know in advance which objects or elements will be used and how they will overlap. We therefore employed a simple object-oriented strategy where we plot each object separately and automatically determine non-overlapping locations to label the used visual elements (Figure 2B). Long variable names are truncated and are displayed as a tooltip if the user hovers over them. An alternative option for generating a legend is manual labelling of one of the generated Glyph Shapes as we did for Figures 3, 4.

### 2.4 Data Normalisation

Like heat maps and other glyph-based approaches, our method requires normalising the data between 0 and 1 so they are mapped to the same scale (Sailem et al., 2015). If the uploaded data is not normalised, then it is automatically scaled. We note that some variables can be related (represent the same scale). For example, if the width and length of an object were scaled independently, their relative ratio will not provide a faithful representation of the actual data. To tackle this problem, we introduce linked variable functionality in the Data Normalisation sub-menu on the right. Linked variables are mapped to the same scale. For instance, if the length of the largest cell is 100 pixels and its width is 60 pixels, then they will be scaled to 1 and 0.6 respectively when defined as linked variables but to 1 and 1 when scaled independently (assuming that this cell is also the widest cell).

### 2.5 Implementation

ShapoGraphy is developed using HTML5 and JavaScript. The shapes and their customisation are implemented using paper.js library. It is a client-side web application which means that all the processing happens at the user end and minimal data is uploaded to our server. This circumvents potential privacy issues.

We defined a portfolio of templates to accommodate different data (Figures 3, 4 and Supplementary Figures S1, S2). The user can choose an existing template to map their data or modify an existing template by adding additional objects and changing shape-data mapping. They can also delete or hide unwanted objects for maximal flexibility.

### 2.6 Import and Export

We offer multiple options for exporting visualisation created in ShapoGraphy including Portable Graphics Format (PNG) or Scalable Vector Graphic (SVG). The latter is particularly useful if the user needs to tweak the design in a graphic editors. The user can export their template which will be saved as a JavaScript Object Notation (JSON) file. This can be then imported using the “Load Project” function from the File menu.

The File menu on top left allows the user to upload data, load demo data or load a project (data file and previously saved templates). If the variable names in the template and variable names in the data file do not match, then the user can remap these variables from the right menu.

### 2.7 Datasets

The datasets used in this manuscript are available as demo files from the file menu in ShapoGraphy.

#### 2.7.1 Wound Scratch Data

Wound scratch data was obtained from an image-based siRNA screen measuring human dermal lymphatic endothelial cells migration into a scratch wound created in a cell monolayer^20^. Cells were imaged at 0 and 24 h following wounding at 4x objective. Cells were detected and the wound area was segmented using DeepScratch^15^. Measurements of wound size and cell numbers at 24 h were normalised to timepoint 0 h and represented using ShapoGraphy.

#### 2.7.2 Multiplexed Imaging Data

Multiplexed imaging data of 2000 HeLa cells was obtained from Gut et al. (2018) where immunofluorescence of different markers was performed in cycles to image the subcellular localisation of 40 proteins^16^. Ten variables were selected to showcase ShapoGraphy. Data was scaled and transformed using UMAP. K-means was used to group phenotypically similar cells into six clusters. The average of UMAP dimension 1 and 2 was calculated for each cluster.

Three cell-shaped objects were created to represent PI3K/AKT/mTOR pathway (pAKT, p4EBp1 and pS6, where “p” denote protein phosphorylation) on the cell periphery as the proportion of symbols overlayed on the object outline (Figure 4C). The grid density in the square surrounding the cell object represents the local cell density. The abundance of late endosomes (CAV1) was represented as “x” symbols filling the cytosol. Golgi and centrosome organelles were abstracted as circles with a colour gradient reflecting their abundance. Three variables were mapped to the circle-shaped nucleus object: the value of nuclear pore protein (NUPS) was mapped to the border of the nucleus object, the level of YAP transcription factor was mapped to the colour of the nucleus object, and the abundance of cell proliferation protein PCNA was represented as dots filling the nucleus object. The position of each Shape Glyph is mapped to the cluster centre using the Positional Mapping sub-menu.

## 3 Results

### 3.1 Case Studies

We created various templates to represent diverse image datasets. These include phenotypic data of breast tumours based on METABRIC study (Curtis et al., 2012) and cell shape data from our PhenoPlot study (Supplementary Figures S1, S2). Here, we discuss in detail the application of ShapoGraphy to multiplexed and wound healing data. Notably all these templates can also be used with any numerical data.

#### 3.1.1 Visualising Scratch Assays Data

As a first use case, we used ShapoGraphy to visualise the effect of gene perturbations on cell migration into a wound scratch (Javer et al., 2020). In this dataset, the closure of an artificially made wound by human lymphatic endothelial cells is measured over a period of 24 h to determine how different gene knockdowns, using siRNA, affect cell migration (Figure 3A). In addition to the change in wound area, we measured the number and area of cells as they can affect the final wound area.

To represent this data using ShapoGraphy, the well and the wound were depicted as rectangles mimicking the shape of the actual measured data. We chose to represent the cell area using the colour of the well object because it applies to most of the cells. We mapped the density of the cells to a mesh density element because they represent a similar concept, i.e., density, and therefore are easier to link. The height of the wound object represents the change in wound area which naturally corresponds to the healing process where cells migrate vertically to close the created wound (Figure 3B). Compared to a bar chart (Figure 3C), such representation reveals more readily that depletion of AKT2 and PLCG1 genes results in a similar wound area and that AKT2 knockdown results in lower cell density and higher cell area than PLCG1. Therefore, their effects on cell motility are not equal. Similarly, depleting CDH5 and CDC42 significantly affects wound area, but CDH5 knockdown results in significantly lower cell number and very large cells suggesting that these two genes affect cell motility through different mechanisms (Figure 3B). This pattern is difficult to discern from raw images as wound measurements need to be normalised to the initial timepoint (0 h) (Figure 3A). A bar chart of these three variables, on the other hand, does not allow for metaphoric association between these variables making it difficult to identify the relationships between them. These results show that ShapoGraphy allows identifying interactions between variables as it provides a more intuitive representation which supports making scientific conclusions from complex phenotypic data.

#### 3.1.2 Visualisation of Multiplexed Imaging Data

Next, ShapoGraphy was used to obtain high data density of single cell phenotypes in multivariate multiplexed imaging data measuring 40 markers (Gut et al., 2018). Multiplexed imaging allows simultaneous imaging of spatial protein activities, subcellular organisation as well as various cell identities (Zhang et al., 2013). Since tens of markers can be imaged, colour coding of the different proteins is no longer useful to visualise this information (Walter et al., 2010). To study the phenotypic heterogeneity of cancer HeLa cells, we analysed data from 2000 cells that were stained with markers highlighting various cellular organelles and signalling components including the AKT pathway (Methods). Using k-means and UMAP cells could be clustered to characterise different subpopulations but the specifics of the underlying phenotypic differences between the clusters could not be obtained (Figure 4A and Methods). Heat maps allow studying all the measured markers individually but require many cognitive calculations such as searching for the different variables and remembering their values to compare them (Figure 4B). This makes them challenging to interpret.

In order to facilitate the understanding of single cell phenotypes that are derived from multiplexed data, ShapoGraphy was used to design a template where the visual elements resemble the represented data attributes. We combined several objects to create a structure that mimics the measured data and depicts the hierarchical nature of bioimage data (Table 2). For example, as cells are composed of multiple organelles, we used different circled objects inside the cell object to represent data of proteins localised to different organelles: nucleus, Golgi and centrosomes. On the cell object, we represented the AKT signalling cascade as consecutive layers on the cell periphery. We created a square around the cell object to represent its context based on local cell density. As in the first use case, we mapped the cell density to the mesh density as they can be easily associated. Symbol filling is well suited for representing endosomal abundance because of its punctate distribution in the cytosol. The rationale for the different design choices is explained in Table 2. This abstract representation of different components in the cell and their spatial arrangement provides a more intuitive representation where the various elements in the Shape Glyph can be easily linked to the measured variables.

**TABLE 2:** List of design decisions (objects and visual elements) used in Figure 3.

Design	Description		
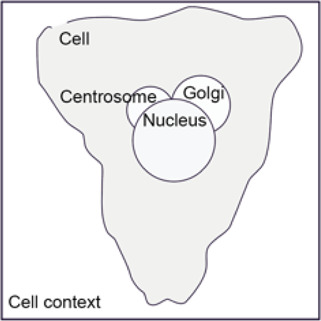	For intuitive mapping of multiplexed image data, we used different objects to create a hierarchy and represent features associated with different cellular compartments		
	Golgi (GM130) 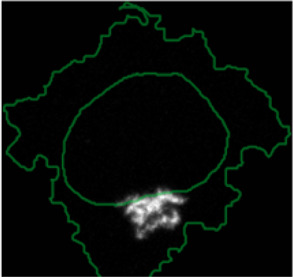	Centrosomes (Pericentrin) 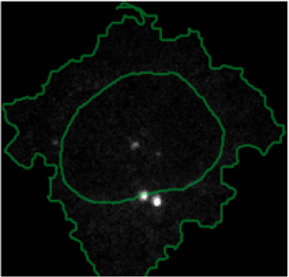	
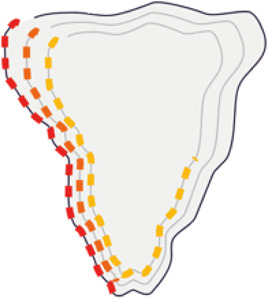	Signalling of AKT is represented as symbols overlayed on the cell object outline. Three cell-shaped objects are layered to represent additional information at the cell periphery. This configuration allows representing the signalling cascade pAKT -> p4EBP1 and pS6. Different colours are used for these different proteins so they can be distinguished easily		
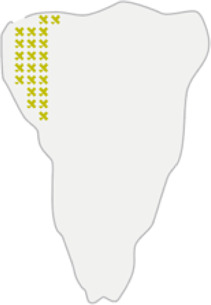	Endosome abundance, based on CAV1, is represented as symbols filling the inner cell-shaped object. This visual channel is well-suited to represent the punctate distribution of endosomes in the cell		
	Caveolin (CAV1) 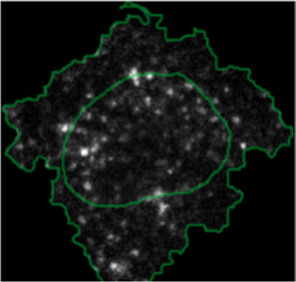		
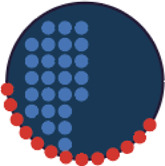	Multiple variables are mapped to the nucleus object. The border symbol (red dots overlying nucleus glyph) provides a faithful representation of nuclear pore protein (NUPS) that localises to the nucleus membrane. The nucleus colour is used to represent the level of YAP transcription factor. While the cell proliferation protein PCNA is represented using symbol filling due to its punctate appearance (blue dots)		
	NUPS 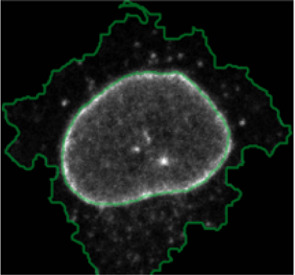	PCNA 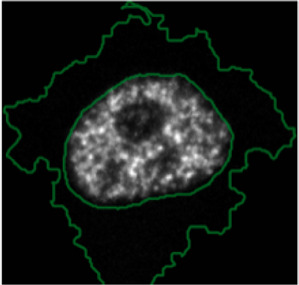	YAP 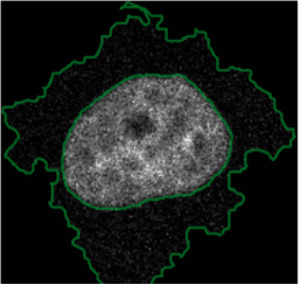
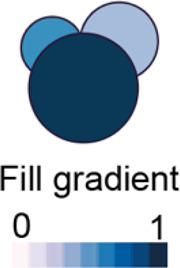	We used colour gradient in a manner similar to a heat map to represent the value of proteins that localise to different organelles. For example, YAP transcription factor is mapped to the colour of the nucleus glyph and Pericentrin is mapped to the colour of the centrosome glyph where they localise. The same colour map is used to enable comparison. The colour provides a good choice when the objects are overlapping, and part of the object is concealed as it is uniform throughout the object		
	YAP (nuclear) 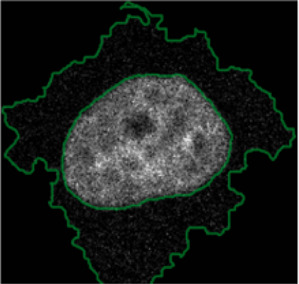	Golgi (GM130) 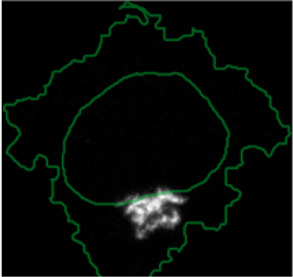	Centrosomes 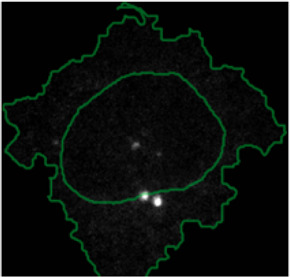

A major advantage of using glyph representations is that the quantitative information is self-contained and therefore the position channel can be used to visualise additional dimensions. We positioned the composite glyphs based on the centre of identified clusters in the reduced UMAP space to help sorting these composite glyphs and comparing cluster phenotypes (Methods).

Figure 4C shows that Cluster 2, 4, and 6 on the right have high cell density (grid density) and low late endosome abundance (x symbols filling the cytosol). Cluster 6 and 4 are highly similar, but Cluster 6 has the highest pS6 levels across all clusters, while Cluster 2 has very high pAKT and p4EBp1, centrosomes (Pericentrin), nuclear pore proteins (NUPS), but low YAP values. Cluster 3 has also high pAKT and p4EBp1 like Cluster 2 but has lower cell density and the highest endosome abundance. Discussing our results with biologists, they found that these representations help them understand their data better as it is easier to identify and relate the differences between clusters to image data. In comparison, Figure 4B depicts the same information in a heat map which can complement our Shape Glyphs but does not help the user to build a mental picture of the data. Therefore, ShapoGraphy provides a more expressive representation of phenotypic classes and their biological relevance based on high dimensional single-cell data which allows scientists to uncover and study complex patterns and relationships in the data.

### 3.2 Guidelines for Designing Glyph-Based Representations Using ShapoGraphy

We reflect on our learning from developing various use cases using ShapoGraphy and our discussions with potential users. First, while the motivation of combining different objects is to create semantically relevant representations, it is possible that some object and/or element combinations can be perceived differently from what is intended or can result in undesirable properties. For instance, using a mesh element on a hierarchy of circles can create geometric patterns (Supplementary Figure S4). Here we propose that ShapoGraphy provides a fast approach for assessing such interactions. Moreover, it allows experimenting with various designs that can inspire new visual representations.

We noticed that when creating composite glyphs, users tried to infer meaning from aspects of the element configuration which were not mapped to data as the user was looking for patterns in the plotted glyphs. This was the case when using the mesh element with random orientation. This problem did not arise when the user learned that this is a static configuration. As object colour can be either statically defined or dynamically mapped to the variable, we recommend using it consistently for all objects. For example, the coloured objects in Figure 4C (Golgi, centrosome, and nucleus) reflect the variable value and the same colour is used otherwise. We also experimented with assigning the same colour for all symbols/elements, however some users found this representation difficult to scan and using different colours helped the user in distinguishing and scanning these distinct elements (Supplementary Figure S3). Continuing the discussion of colour assignment, we found that using the same colour map for “Fill gradient” element is important to make comparisons across different objects easier.

Consideration should be given to the number of features when using Shape Glyphs as our working mental memory is limited and can handle only 5–10 variables at a time (Cairo, 2013). Selection of important features can be achieved through interactive exploration in ShapoGraphy and using the hide/show functionality to identify the most relevant information to be communicated to the reader.

Object occlusion is another aspect that needs to be considered when designing Shape Glyphs where objects are overlayed on top of each other or partially overlap. Visual elements such as colour and mesh density are less affected when part of the object is occluded. For example, the nucleus object lies on the top and occlude part of the Golgi and centrosome objects in Figure 4C, but does not affect the perceived quantitative mapping as colour is uniform throughout the object.

## 4 Discussion

The human brain perceives information by converting visual stimuli to symbolic representations that are then interpreted based on our memories and previous knowledge. Visualisation approaches help our brain create a mental visual image of quantitative data in order to recognise patterns and identify interesting relationships that might be missed otherwise (Tufte, 2001). ShapoGraphy is a new visualisation approach that allows creating bespoke glyph-based representations by constructing composite glyphs that combine different shapes and symbols, each of which encodes multiple variables. To our knowledge, such an approach to data visualisation has not been explicitly proposed before and no tool is available to create such graphical representations automatically.

The main advantage of ShapoGraphy is that it enables the creation of a metaphoric quantitative representation of the data to aid the reader in interpreting, understanding, and communicating scientific results. This makes it perfectly suited for bioimages because of the structural and hierarchical nature of these datasets. Nonetheless, ShapoGraphy is a very versatile tool and can be applied to any numerical data such as single cell RNA sequencing, proteomics, or non biological data. Another advantage of Shape Glyphs is that such pictorial representations can attract more attention from the reader as they stimulate more cognitive activity (Borgo et al., 2013). This can be beneficial when communicating data with a broad audience. Therefore, ShapoGraphy serves as a general-purpose methodology for creating more engaging and intuitive graphic representations.

ShapoGraphy complements existing visualisation methods such as heat maps, t-SNE and UMAPs. While the latter approaches provide a global picture of the major trends or structure in the data, ShapoGraphy allows a more detailed understanding of multiparametric phenotypes. It aims to represent quantitative data so the user can compare different variable values relative to each other, rather than generating an actual picture of the image data. Such distinction is necessary as image data are often normalised which make interpreting raw image data more challenging and subjective. Currently, our approach is best suited for summarising and providing higher information density of major phenotypes in the data, rather than individual data points. This is because the pictorial nature of the generated representations requires high resolution and more space. These phenotypes can be identified using clustering or classification tasks. A potential future direction is to extend our approach to gain multi-level summaries of the data enabling effective visualisation of a larger number of data points.

The high flexibility offered by ShapoGraphy to combine and position different Shape Glyphs and symbols, including hand-drawn shapes, provides an unprecedented opportunity to easily evaluate various designs. This is an important distinction from glyph-based visualisation methods that have been developed for medical images as they provide a very bespoke representation for the problem at hand making them hard to transfer to other types of images (Ropinski et al., 2011). Notably, it can take time to learn new visual encodings representing specific or complex domain knowledge (Borgo et al., 2013). Once learned, such glyph-based visualisations can become more effective for specialised users. Many examples can be found in the genomics domain including representations of gene variants or ideograms of chromosome structure (Wolfe et al., 2013; L’Yi et al., 2022). Redundant or alternative representations, that are more familiar to the user, can be used in parallel with ShapoGraphy when introducing new visual designs (Cairo, 2013).

An important future direction is to perform a user study for evaluating various aspects of glyph-based designs generated by ShapoGraphy. Given the infinite number of designs that can be generated using ShapoGraphy, such a study should be carefully planned and focused on the most recurring element combinations or designs that are most well-received in the community. Moreover, this assessment should align well with the purpose of the visualisation such as facilitating the discovery of complex patterns, communicating with a broad audience, interpretability, or effectiveness. The user study could advance our understanding of how various elements interact with each other and might highlight potential perturbations that can be programmatically employed to improve future versions of ShapoGraphy. For example, multilevel glyphs can be used to minimise occlusion (Müller et al., 2014) or sequential highlighting of certain glyph elements selected by the user. This could also inform practices on visual elements that are most effective when combined and which combinations should be avoided which ultimately could accelerate the development of glyph-based visualisations.

Another interesting extension of ShapoGraphy would be the automation of the mapping between numerical features and shapes. One way to achieve that is to adopt a generative approach where multiple glyph-variable mappings are proposed for the user to choose from. Such an approach could inspire visualisation design (Brehmer et al., 2022). This would greatly improve the user experience as currently, the user needs to map features one by one. We tackle this limitation by enabling users to save their mapping along with their created composite glyph configuration as a JSON file for later use. We also offer a range of templates that can be directly used or adjusted by the user.

To conclude, ShapoGraphy can be used in all steps of data analysis to create intuitive pictorial representations of any data type. It can be used to summarise analysis results obtained from clustering or classification approaches, as well as an educational tool. We believe that the unique flexibility offered by ShapoGraphy will expand our visual vocabularies, accelerate the evolution of glyph-based visualisation, inspire creative design, and stimulate the development of new visual encoding schemas. Most importantly, ShapoGraphy is not restricted to image data but can be applied to any numerical data.

## Data Availability

The original contributions presented in the study are available at www.shapography.com, further inquiries can be directed to the corresponding author.
