# Supplementary material for: ShapoGraphy: A User-Friendly Web Application for Creating Bespoke and Intuitive Visualisation of Biomedical Data
Source: Front Bioinform. 2022 Jul 4;2:788607. doi: 10.3389/fbinf.2022.788607 (PMC9580894; doi:10.3389/fbinf.2022.788607)
Supplement: Supplementary file 1 [file Image1.pdf]

Sup. Fig. 1

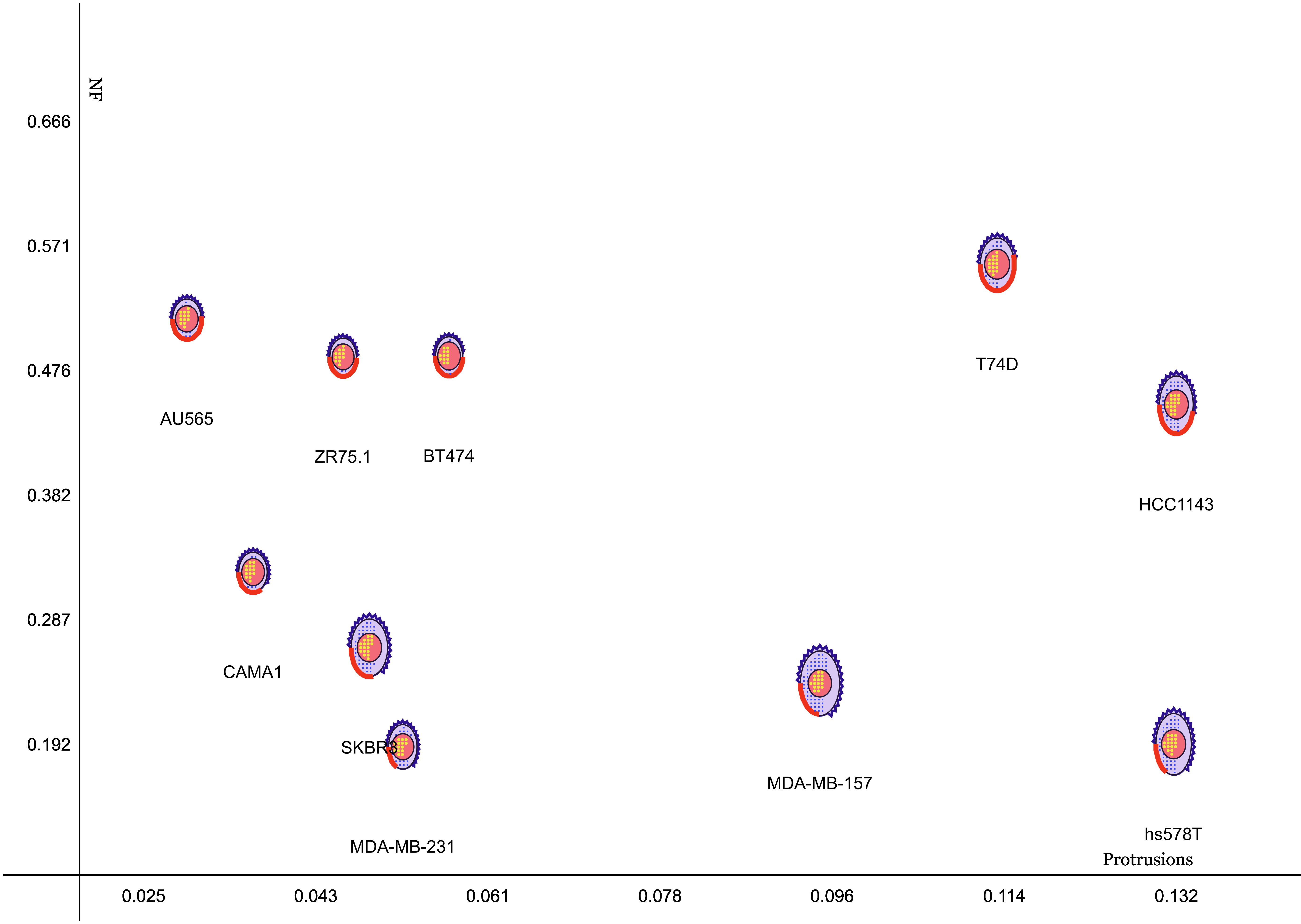

Supplementary Figure 1. Representation of shape information using ShapoGraphy. The morphology of 18 breast cancer cell lines is visualised to represent eight dimensions including cellular and nuclear dimensions, ruffliness (irregularity of cell border), NF (neighbour fraction or the fraction of cell border in contact with other cells), cell texture (the variation of DHE stain in the cytoplasm), nucleus textures (the variation in DAPI intensity in the nucleus). This data is taken from our previous PhenoPlot study (Sailem et al., Nature Communications, 2015).

Sup. Fig. 2

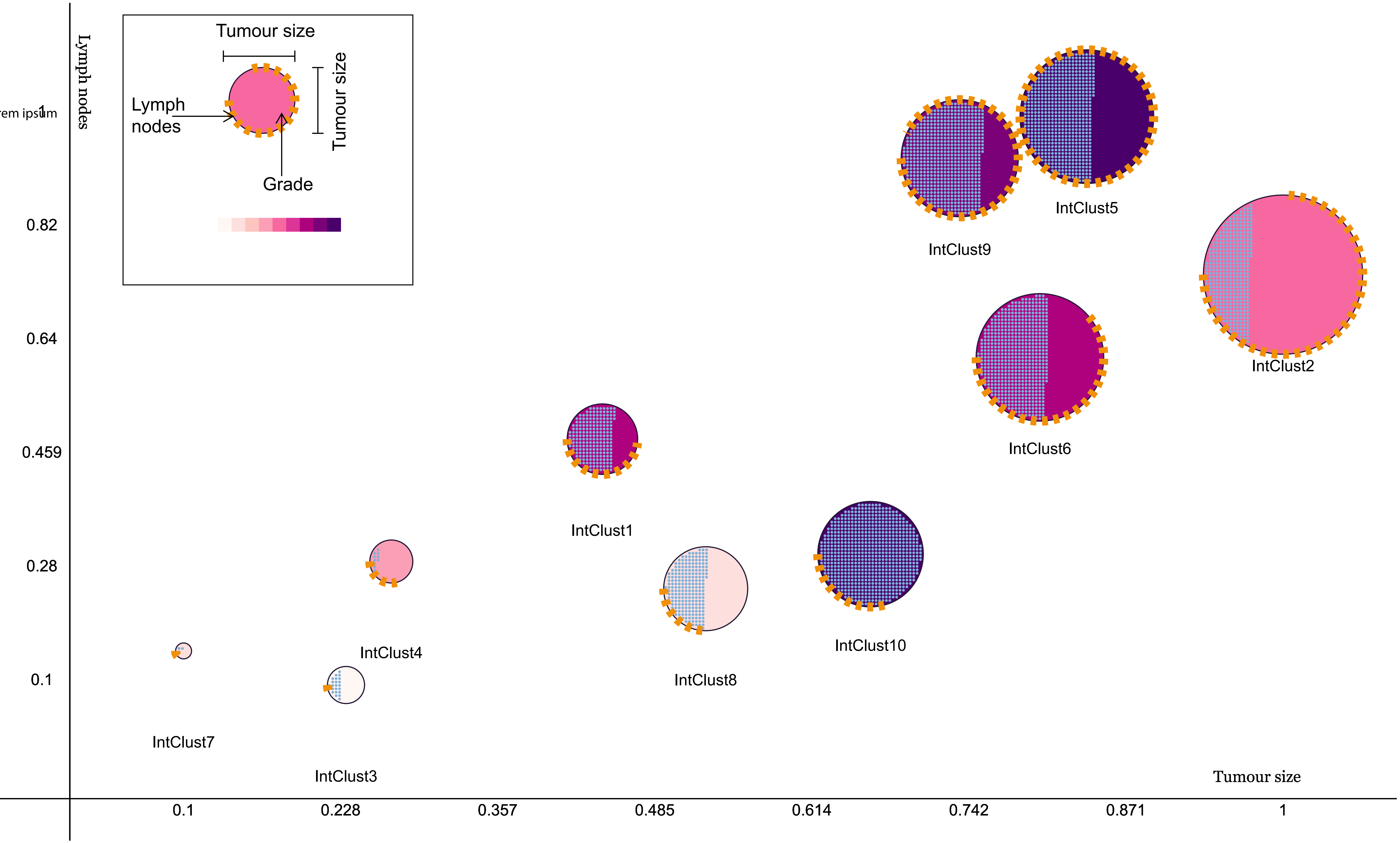

Supplementary Figure 2. Representation of phenotypic features of breast tumour clusters (IntClust) based on genomic and transcriptional data in the METABRIC study (Curtis et al., Nature, 2018). Circle size represents tumour size. The proportion of dots in the tumour represents cellularity (density of tumour cells). Lymph nodes indicate how many lymph nodes the tumour has spread to. The average tumour grade is represented as a gradient.

Sup. Fig. 3

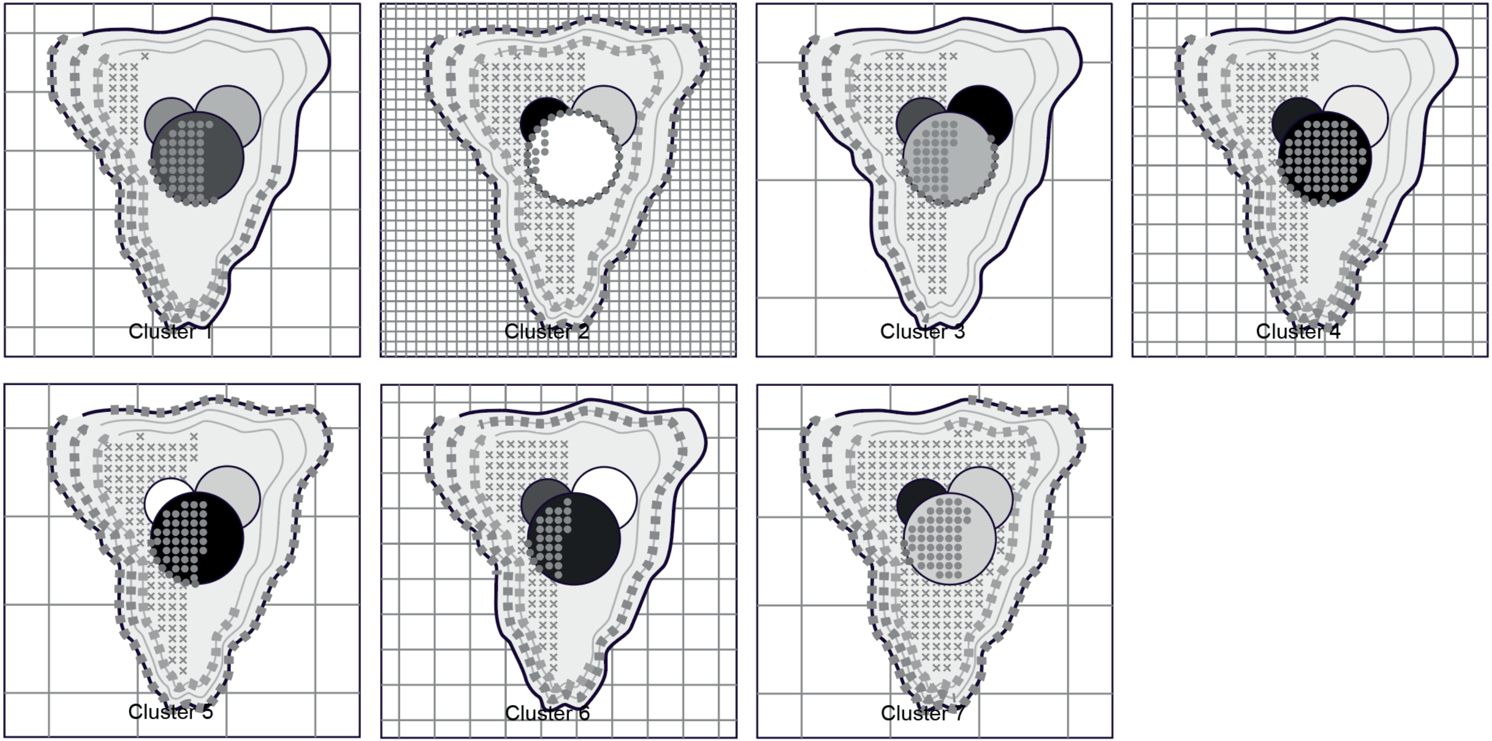

Supplementary Figure 3. Grayscale representation of multiple elements in ShapoGraphy. Shown is the same representation in Figure 4 is shown.

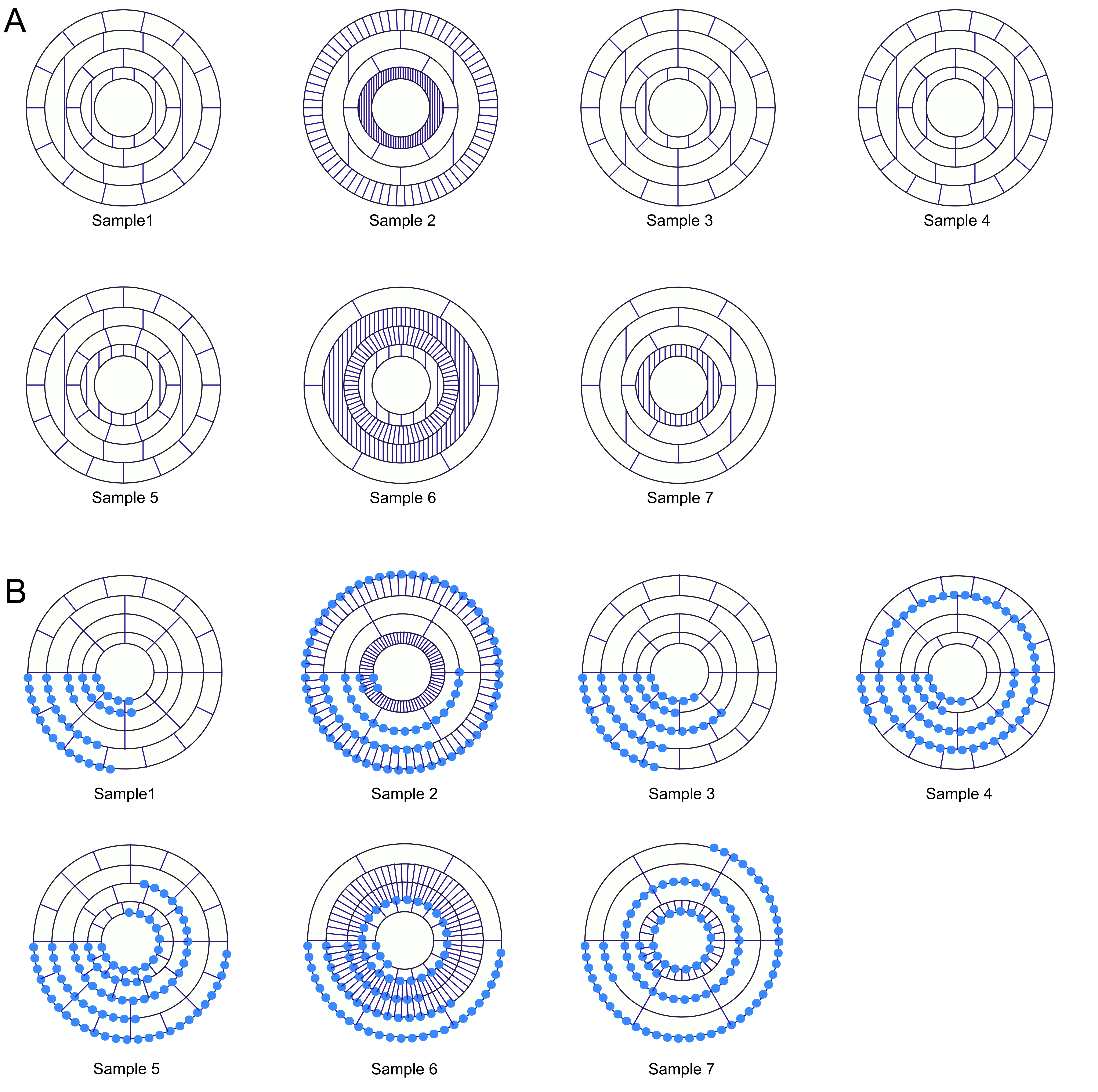

Supplementary Figure 4. Exploration of glyph-based designs using ShapoGraphy. (A-B) Certain element combinations can create unintended patterns or inspire new designs.
